# Medullar Kock without Pott: 13 cases observed at the university hospital center of Conakry, Guinea

**Published:** 2018-10-07

**Authors:** Fode Abass Cisse, Foksouna Sakadi, Nana Rahamatou Aminou Tassiou, Amadou Talibe Balde, Arcel Steven Nitcheu Woga, Aissatou Kenda Bah, Souleymane Djigue Barry, Ibrahima Sory Souare, Mohamed Lamine Toure, Amara Cisse

**Affiliations:** 1 Department of Neurology, University Hospital of Conakry, Guinea; 2 Department of Neurosurgery, University Hospital of Conakry, Guinea

**Keywords:** Myelopathy, Tuberculosis, Guinea

## Abstract

**Background:** The diagnostic certainty of medullar tuberculosis (TB) without Pott disease is difficult to establish in a tropical environment with the large group of infectious, parasitic, and systemic myelopathies, despite the increasing availability of magnetic resonance imaging (MRI) data and improvement of biological exploration platforms.

**Methods:** We retrospectively analyzed the files of 186 patients hospitalized in the Department of Neurology and Neurosurgery of the University Hospital Center of Conakry, Guinea, between 2008 and 2016 for the management of non-compressive and compressive myelopathy. Biological evidence of TB infection was demonstrated for 13 (6.9%) patients.

**Results:** Infectious clinical picture prior to the development of neurological signs was reported in 11 patients (84.6%). The neurological signs were summed up by the existence of a sensitivo-motor semiology of progressive evolution (100% of cases) with sphincter disorders in 11 patients (84.6%) and a medullary compression symptomatology with a lesion and under lesion syndrome from the outset in 4 patients (30.8%). Medullary MRI revealed an extensive intramedullary hypersignal in 9 patients with non-compressive myelopathy and in 4 cases, the lesions appeared in T1 hypersignal and T2 isosignal were localized. Lumbar puncture (LP) revealed lymphocytic pleocytosis, hypoglucorrhage (0.3 to 0.5 g/l), and leukocytosis.

**Conclusion:** This study reveals a classic clinical, biological, neuroradiological, and evolutionary profile of compressive and non-compressive myelopathies. These results are important for the therapeutic and evolutionary discussion of TB myelopathies for good management.

## Introduction

Despite the increased incidence of extra pulmonary forms of tuberculosis (TB) in both immunocompetent and immunosuppressed patients,^1^^-^^4^ medullar localizations without Pott disease are rarely reported.^2^^-^^5^

It is well established in several old and new works that TB myelopathies are often related to lesions with Pott disease;^1^^,^^6^^-^^9^ however, isolated medullary forms are poorly described, whereas central nervous system (CNS) TB accounts for 0.5% to 3.6% of TB cases.^4^^,^^5^^,^^10^

We report 13 cases of TB myelopathies without Pott disease for the purpose of a reevaluation of this pathology from the clinical, neuroradiological, and evolutionary point of view.

The interest of this work resides in the fact that these observations illustrate the primitive medullary lesions of TB origin without osteo-vertebral lesions and the diagnostic difficulties that they comprise in the vast group of acute and chronic myelopathies in Sub-Saharan Africa.^11^

## Materials and Methods

Thirteen patients have been observed retrospectively between January 1^st^, 2008, and December 31^st^, 2016, in the Departments of Neurology and Neurosurgery of the Conakry University Hospital Center, Guinea, the only center in the country for the specialized care of patients suffering from neurological and neurosurgical diseases.

All patients benefited from a series of complementary examinations including:

Blood count and sedimentation rate (SR), fasting blood glucose, 24-hour proteinuria, ionogram, serum calcium, serum iron, serum glutamic pyruvic transaminase (SGPT) and serum glutamic oxaloacetic transaminase (SGOT), creatine phosphokinase (CPK), c-reactive protein (CRP), rheumatoid factor (RF);

Analysis of cerebrospinal fluid (CSF) by lumbar puncture (LP), performed in 9 (69.2%) patients allowing cytological and biochemical evaluation (proteinorrachie, glycorrhachia, chlorurorrachia);

Direct bacteriological examination and culture with BK research and polymerase chain reaction (PCR). 

As part of the differential diagnosis of the other etiologies of myelopathy, the following examinations were performed:

PCR, Epstein-Barr virus (EBV), human immunodeficiency virus (HIV), enterovirus;

Serologies HIV, human T-cell leukemia virus type 1 (HTLV-1), varicella-zoster virus (VZV), venereal disease research laboratory-Treponema pallidum hemagglutination assays (VDRL-TPHA), enterovirus;

Bilharzial serologies, hydatidosis, toxoplasmosis, trichinosis, cysticercosis in blood, and CSF.

X-ray of the thorax was requested for all patients. Magnetic resonance imaging (MRI) has been focused on the entire cervico-dorsal and lumbar spinal cord with T1-weighted sagittal and axial sections before and after gadolinium injection and T2-weighted.

Four patients underwent biopsy after laminectomy and excision of the granuloma.

All patients received treatment with Rifampicin, Isoniazid, Pyrazinamide, Ethambutol (4RHZE) for 2 months followed by dual RH therapy for 8 to 14 months according to the World Health Organization (WHO) recommendation.^1^ Corticosteroid therapy has been instituted for 4 to 6 weeks.

The evolution was evaluated according to the criteria enacted in the Lipton and Taesdall Scale^11^ in 3 stages:

Stage 1: Poor prognosis with impossible walking or walking distance of less than 100 m, permanent anesthesia, partial sphincter control or self-contained bladder.

Stage 2: Average prognosis that may be associated with walking discomfort requiring help with permanent sensory signs and inconstant disorders of sphincter control.

Stage 3: Good prognosis involving normal walking, absence of sphincter disorder or rare urinary urgency, normal neurological examination or minimal signs.

## Results

From January 2008 to December 2016, 186 patients (9.2%) from all neurology and neurosurgery admissions were hospitalized for non-traumatic paraplegia or quadriplegia, including 9 patients with progressive myelopathies and 4 patients for spinal cord compression with lesion and under lesion syndrome of TB origin.

The average age was 29.6 years ranging from 16 to 58 years. Hyperthermia above 39 °C associated with a flu-like syndrome with asthenia and fatigue were observed in 84.6% of patients before the appearance of neurological semiology. Antecedents were sought in all patients with a subacute onset (at least 7 days) in 3 (23.1%) cases. In 2 (15.4%) cases, the diagnosis upon hospitalization was bilharzia medullary compression, since these two patients came from a recognized high bilharzia endemic region with indication of bathing in a river. The clinical signs at the start and state stage are summarized in table 1.

**Table 1:** Clinical pictures at the beginning and state phase (n = 13)

Clinical signs at beginning phase	n (%)
**Hyperthermia (fever)**	11 (84.6)
**Night sweat**	6 (46.2)
**Local cervical, dorsal or lumbar pain**	11 (84.6)
**Numbness and tingling of the lower limbs**	6 (46.2)
**Muscle cramps**	2 (15.4)
**Lameness**	6 (46.2)
**Alteration of general condition (physical asthenia and fatigue)**	7 (53.8)
**Disorders of consciousness**	1 (7.7)
**Clinical signs of the state phase**	
**Motor/sensory/vegetative syndrome (paraplegia, paraparesis, tetraplegia, tetraparesis, sensory level)**	13 (100)
**Sphincter disorders (dysuria, acute retention, constipation)**	11 (84.6)
**Disorders of vigilance (confusional syndrome)**	3 (23.1)
**Pure motor aphasia**	1 (7.7)

The clinical picture at the beginning phase was dominated by pyramidal syndrome (84.6%) and infectious syndrome (84.6%). Motor/sensory/vegetative syndromes dominated the state phase. In one patient, it was associated with pure motor aphasia. Eleven (84.6%) patients had sphincter disorders. Four (30.8%) patients had secondary locations: pulmonary (3) and cerebral (1).

**Figure 1:**
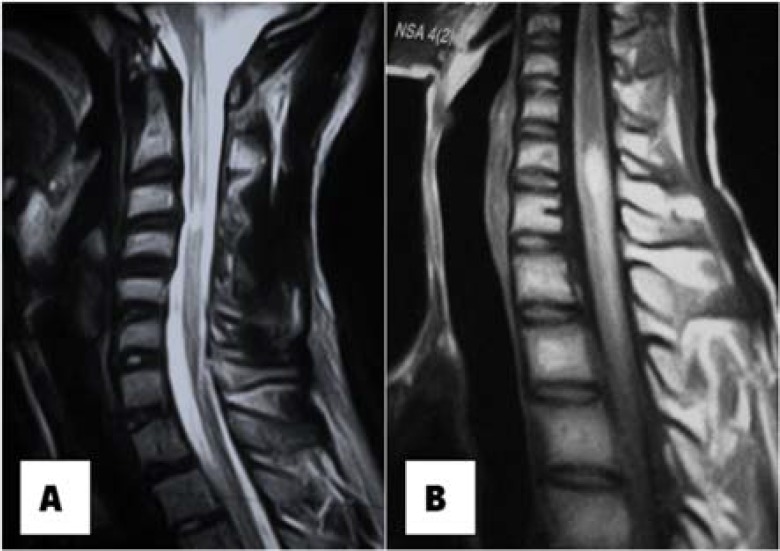
Magnetic resonance imaging (MRI) in T1 sequence with injection, carried out after 1 month of symptomatology, objective a hypersignal opposite C1-C5 (A), MRI in T2 sequence without injection performed after 2 months of its symptomatology, objective a hypersignal opposite C3-C6 (B)

***Neuroradiological data:*** MRI showed hypersignal or hyposignal heterogeneous images with injection T1 and T2 sequences in 69.2% of cases, reflecting a conglomeration of microgranulomas with an average consultation time of 96.1 days (Figure 1). In 30.8% of the cases, the images were in rings or cockades with a hyposignal surrounded by hypersignal leading to spinal compression with an average consultation time of 191.8 days (Table 2).

***Biological data:*** Table 3 presents the particularities of the assessments made.

Pathological findings showed inflammatory reactions with epithelioid and gigantocellular granulomas in 2 cases, caseous necrosis in 1 case, and caseum in 1 case.

9 patients were directly treated by the TB chemotherapy after confirmation of the diagnosis. 4 patients were referred to neurosurgery for an intervention followed by medical treatment on our premises.

In general, under TB chemotherapy and corticosteroids (solumedrol) in bolus then orally, the evolution was favorable in 46.2% of cases and stationary in 38.5% of cases according to the Lipton and Taesdall score at 12 months. 1 (7.7%) patient died at 3 months of follow up, the other patient was lost to follow up after 6 months. Long-term assessment was not possible in stationary patients as they were lost to follow up.

The evolutionary profile is summarized in table 4.

## Discussion

TB remains and stays a real public health problem in Sub-Saharan African countries in both pulmonary and extrapulmonary forms.^1^^,^^11^

In Guinea, the epidemiological situation as described in the 2016 health statistical yearbook, notes for 1397 TB cases with an incidence rate of 0.13 per 1000 population.^12^ However, these figures are far from representing the reality, because of the under-reporting resulting from the under-medicalization and expression of the insufficiency of the means of TB detection.

**Table 2:** Epidemiological neuroradiological and therapeutic characteristics (n = 13)

**Patient**	**Age/sex**	**Antecedent**	**Time ** **(day)**	**Site**	**Sequence**	**Gadolinium**	**Lesion**	**Association**	**Treatment**
1	26/Male	Concept of contage	78	D8-D10 Intramedullary	T1	+	Hypersignal	Pulmonary	Medical
2	29/Male	Without antecedent	105	C4-C5 Intramedullary	T2	-	Hypersignal	-	Medical
3	32/Female	Concept of contage	91	D5-D7 Intramedullary	T2	-	Hyposignal	-	Medical
4	34/Male	Without antecedent	126	D2-D6 Intramedullary	T2	-	Ring hypersignal	Pulmonary	Surgical
5	35/Male	PTBT	112	D12-L2 Intramedullary	T1	+	Hypersignal	-	Medical
6	42/Female	Concept of contage	98	C1-C5 Intramedullary	T1	+	Hypersignal	Cerebral	Medical
7	46//Female	Without antecedent	209	D2-D6 Intramedullary	T1	+	Ring hypersignal	Pulmonary	Surgical
8	49/Male	TPTBC	98	C3-C5 Intramedullary	T2	-	Hypersignal	-	Medical
9	49/Female	Without antecedent	175	D8-D9 Intramedullary	T1	+	Ring hypersignal	-	Surgical
10	55/Female	PTBT	257	C5-D2 Intramedullary	T1	+	Ring hypersignal	-	Surgical
11	58/Male	TPTBC	102	D3-D4 Intramedullary	T2	-	Hypersignal	-	Medical
12	46/Female	PTBT	88	D11-L1 Intramedullary	T2	-	Hypersignal	-	Medical
13	16/Male	Without antecedent	93	C4-C6, D6-D7 Intramedullary	T1	+	Hypersignal	-	Medical

PTBT: Pulmonary tuberculosis treated but not cured; TPTBC: Treated pulmonary tuberculosis declared cured

This study reports 13 cases of TB myelopathies in the Departments of Neurology and Neurosurgery of the University Hospital Center of Conakry.

These are all suspect cases whose diagnosis was made in the neurology and imaging departments of Conakry University Hospital Center.

**Table 3:** Biological characteristics of tuberculous (TB) acute myelitis (n = 13)

**Patient**	**KB**		**EF (T. ** **Bouchut)**	**Blood**				**LCR**				**Proteinuria ** **(mg/24 ** **hours)**
	**Sputum**	**LCR**		**IDR ** **(mm)**	**CRP ** **(mg/l)**	**SR ** **(mm/hour)**	**PCR**	**L ** **(mm** ^3^ **)**	**P ** **(g/l)**	**G ** **(g/l)**	**PCR**	
1			-					102	1.80	0.42	-	150
2	-	-	+					135	1.40	0.48	-	80
3	+	+	-	25	26	36/42	-	195	1.32	0.39	-	50
4	-	┴	-	28	30	22/48	-	┴	┴	┴	┴	200
5	-	-	-	25	18	18/26	+	186	1.63	0.42	+	75
6	+	+	-	15	24	23/44	-	225	0.86	0.50	+	60
7	+	┴	-	28	22	22/62	+	┴	┴	┴	┴	120
8	-	+	-	20	40	18/36	┴	100	1.28	0.52	+	70
9	-	┴	-	19	10	20/42	+	┴	┴	┴	┴	50
10	-	┴	-	22	6	14/24	+	┴	┴	┴	┴	80
11	-	-	-	15	14	12/24	┴	160	0.75	0.60	+	100
12	-	-	-	20	24	24/28	+	104	1.12	0.45	-	195
13	-	+	+	22	29	22/43	-	157	1.35	0.49	-	75

IDR: Intra-dermo-reaction; CRP: C-reactive protein; PCR: Polymerase chain reaction; LCR: Ligase chain reaction; EF: Eye fundus; KB: Koch bacillus; SR: Sedimentation rate; L: Lymphocytes; P: Proteinorrachie; G: Glycorrhachia

**+**: Present; **-**: Absent; **┴**: Not realized

**Table 4:** Evolutionary profile

**Patient**	**Short term**			**Long term**
	**3 months**	**6 months**	**12 months**	
1	Flasco-spastic paraplegic hypoesthesia going back to D9, stage I	Spastic paraparesis, partial urinary control, stage II of Lipton score	Normal walking total urinary control, sensitivities preserved, stage III	Favorable evolution
2	Flasco-spastic quadriplegia, urinary continence, hypoesthesia going back to C5-C6, stage I	Flasco-spastic tetraparesis, total urinary control, conserved sensitivities, stage II	Normal walking, stage III	Favorable evolution
3	Flaccid paraplegia, urinary continence, hypoesthesia going back to D6, stage I	Flasco-spastic tetraparesis, partial urinary control, disturbed deep sensibility, stage I	-Spastic paraplegia, partial urinary control, disturbed deep sensibility, stage I	Lost in view
4	Spastic paraplegia, hypoesthesia dating back to D6, stage I	Lost in view	-	-
5	Flaccid paraparesia, retention of urine, sensitivities preserved, stage I	Walking using crutches, stage II	Stage II	Lost in view
6	Spastic quadriplegia, urinary incontinence, hypoesthesia dating back to C3, stage I	Spastic quadriplegia, deep sensitivities disturbed, stage II	The same	Lost in view
7	Spastic paraplegia, urinary retention, hypoesthesia dating back to D6, stage I	Spastic paraplegia, dysuria, diminished surface sensibility decreased D4, stage II	Spastic paraparesis, stage II	Lost in view
8	Flasco-spastic quadriplegia, spastic tetraparesis, hypoesthesia going back to C6, stage I	Spastic tetraparesis, profound sensibility disturbed, stage II	Normal walking, deep sensibility preserved, stage III	Favorable evolution
9	Flaccid paraplegia, urinary retention, hypoesthesia dating back to D9, stage I	Spastic paraparesis, urinary control, stage II	Normal walking, stage III	Favorable evolution
10	Flasco-spastic quadriplegia, urinary incontinence, hypoesthesia back to D3, stage I	Flasco-spastic quadriplegia, hypoesthesia going back to D3, stage I	Same sensitivities preserved, stage I	Lost in view
11	Spastic paraplegia hypoesthesia dating back to D4, stage I	Same disruption to the deep sensitivity, stage I; normal walking preserved sensibility, stage III	Normal walking preserved sensibility, stage III	Favorable evolution
12	-Flaccid paraplegia hypoesthesia going back to L1, stage I	Spastic paraparesis, stage II	Stage III	Favorable evolution
13	Deceased	-	-	Evolution -

All patients were included in this study according to the semiological orientation: non-compressive (9 cases) and compressive (4 cases) TB myelopathies except for all osteo-vertebral lesions (Pott disease).

During the study period, apart from the 13 cases described in this study, 37 cases of classical forms of secondary extension of vertebral TB, descending extension of TB meningitis, and even as primary TB lesion were excluded.

Pathophysiologically, two hypotheses are usually proposed to explain the occurrence of TB myelopathies. In most cases, they would result in the spread of a vertebral focus^1^^,^^3^^-^^5^ or in exceptional cases,^2^^,^^10^ they remain associated with multidrug-resistant pulmonary meningitis, especially in tropical environments.^3^^,^^11^

The so-called isolated myelitis is thus meningomyelitis with concomitant presence of lesions and clinical signs of spinal arachnoid and spinal cord involvement. On the other hand, the observed arachnoid involvement is confirmed by liquid signs of spinal blockage.

TB meningoencephalitis may appear immediately with any treatment.

In general, the medullary involvement of TB is most often secondary to a vertebral location: classical Kock bacilli’s spondylodiscitis or Pott disease.^7^

Medullary TB is rare and constitutes only 0.2% to 5% of all CNS localizations. Its frequency is 2 in 100000 cases of TB and 2 in 1000 cases of neuro-TB.^2^^,^^10^^,^^13^

Data from the literature concerning intramedullary TB are rare and involve very small series ranging from 1 to 33 cases.^2^^-^^5^^,^^14^

Clinical symptomatology does not differ from other medullary processes.^2^ It is most often a medullary compression chart: the onset is usually unilateral or asymmetrical, by the installation of a radicular symptomatology which leaves room in 2 to 4 months for a paraplegia more or less complete with sphincteric disorders.^2^^,^^5^

Clinical neuro-infectious and biological signs are inconsistent.^2^ However, the associated general signs (deterioration of the general state, asthenia, nocturnal sweating, and hyperthermia) and the presence of a second TB localization are of great diagnostic value (Table 2) as well as the existence of antecedents or active TB,^3^ but their absence does not eliminate this pathology as has been reported in some of our patients.

The tropical context would explain the disruption of the inflammatory balance, which is often normal in other studies.^15^ The positivity of TB tests is of real value only if the subject is an unvaccinated child or young adult.^2^ Immune status seems to be a risk factor, this has been widely observed in recent years especially with the advent of HIV infection.^2^^,^^4^ The peculiarity of our patients was that none of them had positive HIV serology.

The diagnosis must be evoked in front of the radiological aspect in an evocative context. MRI is the examination of choice because of its better sensitivity and specificity for the study of the spinal cord.^2^^,^^5^^,^^13^^-^^15^ MRI features vary according to the stage of the lesion.^14^ The typical appearance shows a circular image with hyposignal T1 at the center of the lesion and hyperintensity in T2, with a uniform contrast enhancement in the ring, sometimes associated with a raising of the meninges (Table 2).^2^^,^^14^ However, this aspect is not pathognomonic; hence, there is a need to take into account the clinical epidemiological context and the therapeutic response to make the diagnosis after eliminating the differential diagnosis of spinal cord lesions such as other infectious or granulomatous bone marrow lesions (abscess, sarcoidosis, cysticercosis, syphilis), tumors (gliomas and ependymomas), and lymphomatous or demyelinating disease (multiple sclerosis).^4^^,^^5^^,^^10^

The biopsy performed in 4 operated patients showed inflammatory reactions with epithelioid and gigantocellular granulomas and necrosis in some cases. These anatomopathological aspects are identical to certain cases encountered.^3^^-^^5^

Therapeutic management remains difficult and controversial.^2^^,^^4^^,^^10^^,^^13^ Many authors agree that there is a good response to standard anti-TB medical treatment with 4RHZE quadri-therapy and reserve surgery in case of failure or diagnostic uncertainty.^10^ Optimal treatment seems to be associated with quadri-therapy for three months and then dual therapy for 10 to 12 months.^1^^,^^3^^,^^5^^,^^10^^,^^13^^,^^16^ The use of corticosteroids is not preferred because they have not been shown to be effective.^10^^,^^13^ However, their action on perilesional edema and the improvement of the neurological disorders observed in some patients justify their prescription.^13^

The early initiation of anti-TB chemotherapy will result in a better management of intramedullary TB, even if the diffusion of the molecule into the marrow is slowed down.

## Conclusion

Although intramedullary TB has been poorly described in the world, it poses a public health problem, since it increases the risk of disability. The avenue of MRI has revolutionized the diagnostic management of pathology. The biopsy remains the essential element to make the diagnosis, but the therapeutic response is not left behind.

## Notes:

**How to cite this article:** Cisse FA, Sakadi F, Aminou Tassiou NR, Balde AT, Nitcheu Woga AS, Bah AK, et al. Medullar Kock without Pott: 13 cases observed at the university hospital center of Conakry, Guinea. Iran J Neurol 2018; 17(4): 167-73.
